# Response to Higashi et al.

**DOI:** 10.1186/s40981-019-0272-8

**Published:** 2019-08-13

**Authors:** Kuniaki Moriwaki, Kenji Kayashima

**Affiliations:** 1 Department of Anesthesia, Japan Community Health Care Organization, Kyushu Hospital, 1-8-1 Kishinoura, Yahatanishi-ku, Kitakyushu, Fukuoka 806-8501 Japan; 2 Present address: Department of Anesthesiology, University of Occupational and Environmental Health, Kitakyushu, Fukuoka 807-0804 Japan

To the Editor,

We thank Higashi et al. [1] for their expert opinions on our case report [2]. Herein, we address the concerns raised by them.

We administered neostigmine and atropine in our patient after insufficient sugammadex reversal, although we already used bibliographically sufficient doses of sugammadex (more than 1 mg/kg) to eliminate the possibility of muscle relaxation rebound [3]. We agree with the opinion that intensive care and careful observation are necessary after neostigmine administration in such patients. We monitored the physical status of the patient for 24 h after extubation, although the data were not shown in the article [2]. Neostigmine reversal is associated with a dose-dependent increase in the risk of respiratory complications. However, proper use of neostigmine guided by neuromuscular transmission monitoring results can help eliminate postoperative respiratory complications associated with the use of the neuromuscular-blocking agent [4]. We are not certain about the differences in outcomes between sugammadex and neostigmine in such patients. Notably, sugammadex administration following laparoscopic sleeve gastrectomy has no advantages over neostigmine administration in terms of residual curarization and respiratory complications [5], and limited studies have investigated the effects of sugammadex on postoperative pulmonary outcomes [6].

Mild hypothermia possibly played a role in the prolongation of the neuromuscular blockade in our case, and the pharyngeal temperature was 34.8 °C at the beginning of the operation and 35.4 °C just before extubation. A decrease in body temperature from 36.5 to 34.5 °C increased the duration of action of vecuronium and rocuronium from 28 to 62 min and the spontaneous recovery time from 37 to 80 min [7]. An unexpected decrease in rocuronium elimination due to hypothermia possibly prolonged the neuromuscular blockade [7, 8]. However, under mild hypothermia, deep rocuronium-induced neuromuscular blockade was safely reversed by sugammadex [9], and the efficacy of neostigmine was maintained [7].

Higashi et al. suggested that mechanisms underlying the residual blockade might be complicated and should be further investigated [1]. In addition to hypothermia, rocuronium and magnesium sulfate can affect acetylcholine release. Rocuronium, a non-depolarizing neuromuscular blocker, has pre-synaptic inhibitory effects on acetylcholine release [10]. Magnesium sulfate also shows a pre-synaptic effect by inhibiting acetylcholine release at motor nerve terminals, which may be responsible for the interaction of magnesium sulfate with vecuronium, a non-depolarizing neuromuscular blocker [11, 12]. Increased extracellular magnesium levels decrease the magnitude of acetylcholine-evoked responses and the single-channel conductance of nicotinic acetylcholine receptors at the mouse endplate [12]. Furthermore, magnesium sulfate shows concentration-dependent neuromuscular blocking effects in clinical settings [2].

Hypothermia, along with pre-synaptic inhibitory effects of magnesium sulfate and/or calcium channel antagonists, could have prolonged the neuromuscular blockade in our patient. We should bear in mind that non-depolarizing neuromuscular blockers and magnesium sulfate show pre-synaptic effects by inhibiting acetylcholine release to prolong neuromuscular blockade and cause insufficient reversal even after sugammadex administration. Careful observation after extubation is necessary for patients with prolonged neuromuscular blockade.

## Data Availability

Not applicable.
